# Anodal tDCS augments and preserves working memory beyond time-on-task deficits

**DOI:** 10.1038/s41598-021-98636-y

**Published:** 2021-09-27

**Authors:** Rohith Karthikeyan, Meredith R. Smoot, Ranjana K. Mehta

**Affiliations:** 1 Department of Mechanical Engineering, Texas A&M University, College Station, TX 77840 USA; 2 Department of Electrical and Computer Engineering, Texas A&M University, College Station, TX 77840 USA; 3 Department of Industrial and Systems Engineering, Texas A&M University, College Station, TX 77840 USA

**Keywords:** Neuroscience, Psychology

## Abstract

Transcranial direct current stimulation (tDCS) of the left dorsolateral prefrontal cortex (DLPFC) has been shown to promote working memory (WM), however, its efficacy against time-on-task-related performance decline and associated cognitive fatigue remains uncertain. This study examined the impact of anodal tDCS of the left DLPFC on performance during a fatiguing visuospatial WM test. We adopted a repeated measures design, where 32 healthy adults (16 female), underwent anodal, control and sham tDCS on separate days. They completed an hour long two-back test, with stimulation intensity, onset, and duration set at 1 mA, at the 20th minute for 10 minutes respectively. Task performance, subjective responses, and heart rate variability (HRV) were captured during the experiment. Anodal tDCS substantially improved WM relative to sham tDCS and control in both sexes. These benefits lasted beyond the stimulation interval, and were unique across performance measures. However, no perceptual changes in subjective effort or fatigue levels were noted between conditions, although participants reported greater discomfort during stimulation. While mood and sleepiness changed with *time-on-task*, reflecting fatigue, these were largely similar across conditions. HRV increased under anodal tDCS and control, and plateaued under sham tDCS. We found that short duration anodal tDCS at 1 mA was an effective countermeasure to *time-on-task* deficits during a visuospatial two-back task, with enhancement and preservation of WM capacity. However, these improvements were not available at a perceptual level. Therefore, wider investigations are necessary to determine “how” such solutions will be operationalized in the field, especially within human-centered systems.

## Introduction

Fatigue in the emergency response (ER) domain has been typified as an accepted hazard of the job, with extended work hours, cognitive and physical demands, and sleep deprivation^1^. However, its impact on worker health and safety demands wider attention on contributory neurophysiological mechanisms and countermeasures. Performance decrements due to fatigue endanger not only the personnel involved, but also the public they serve^2^. Firefighters and emergency medical technicians, individuals in occupations with high fatigue-risk, are required to multitask using information from concurrent visual and auditory sources, where their working memory (WM) is frequently a key determinant of task success^3^. This ability can be significantly undermined in the field, for example, wild-land firefighters report sleep deficits exceeding $\approx 30$ hours each week, which severely degrades their situational awareness and decision-making^4^. Schmiechel et al. found that the mere perception of fatigue can impede executive function^5^. Therefore, multimodal solutions to augment or otherwise preserve WM, beyond the subjective experience of fatigue, would bring great value to the operations of all first responders.

Neuroimaging studies have shown that Brodmann areas 46 and 9, i.e. primarily the left dorsolateral prefrontal cortex (DLPFC)^6^, are most associated with WM and related executive function, and therefore, this region has been a focal point for targeted neuromodulation^7^. Transcranial direct current stimulation (tDCS), a non-invasive brain stimulation technique, was shown to improve cortical excitability in those regions and in turn promote WM in healthy adults^6^. Studies mainly report a polarity-dependent effect of tDCS on WM, where anodal tDCS of the left DLPFC with intensity $\in [1,~2]$ mA enabled performance enhancements across multiple WM tests^8–11^. However, recent meta-analyses identified inconsistencies in the effectiveness of tDCS^12–14^ owing to small effect sizes, heterogeneous stimulation parameters (e.g., timing, intensity, or duration) and study designs (e.g. repeated vs. between subjects design) resulting in variability of the observed impacts on accuracy, response time and other WM performance measures. Some evidence also questions the efficacy of sham tDCS as a control paradigm due to its effect on behavioral and physiological responses^15^. These discrepancies demand wider investigations into the effectiveness of tDCS with equity in participants and their experiences.

Of particular interest to us is the intersection of WM, cognitive fatigue, and neurostimulation. In examining the efficacy of tDCS as a fatigue countermeasure, McIntire et al.^16^ demonstrated that anodal tDCS for 30 min at 2 mA resulted in improvements on attentional accuracy during vigilance tasks beyond the effects of sleep deprivation. However, they found no direct effect of stimulation on WM during those experiments. Similarly, Borragan et al. report that tDCS at 1.5 mA for 25 min did not counteract the behavioral influence of cognitive fatigue during a sustained WM paradigm^17^. Therefore, while the benefits of stimulation toward vigilance or attention capacity during fatiguing tasks have been reported^16^, evidence for the same effect on WM remains lacking. Hence, research that evaluates the effectiveness of dosage, timing, and duration in task contexts beyond those previously tested is needed, as these factors drive subsequent use and acceptance in time-sensitive field applications such as ER.

In our previous investigation, we explored the role of tDCS on sustained attention during a prolonged (60 minute) psychomotor vigilance test (PVT)^18^, where we relied on the PVT to serve both as a *fatigue induction* paradigm and as a measure of attention capacity—informed by related efforts that consider the influence of *time-on-task* and its impact on sustained attention^19^. The *time-on-task* effect is analogous to fatiguing protocols in the literature^20,21^, where sustained cognitive demand at fixed or varying workload levels is shown to elicit increases in subjective and objective fatigue indices. We found that anodal tDCS at 1 mA for 10 minutes enhanced vigilance capacity on the PVT beyond this *time-on-task* effect, while response time and accuracy were both seen to decrease otherwise. These findings were encouraging as they addressed the immediacy demands that are characteristic of the ER domain by showing the potential benefits of online tDCS on attention networks notwithstanding task demands that were present and continuing leading into the stimulation interval. Furthermore, the efficacy of a relatively short stimulation interval (10 min) at low current intensity (1 mA) is particularly relevant toward fieldability requirements in emergency response, where work conditions demand unobtrusive and expeditious modes of intervention. In the current study, we build on this investigation to consider the role of stimulation on *time-on-task* related deficits during a WM exercise. Under sustained periods of cognitive workload, human WM is compromised by *time-on-task* related fatigue^22^. This fatigue can manifest as an increase in response time, a decrease in accuracy, an increase in self-reported fatigue scores or a decrease in motivation to continue task performance^22,23^. Previous investigations have successfully validated the use of a *two-back* protocol as a *fatigue induction* paradigm, specifically the work by Shigihara and Tanaka et al.^24,25^ employed a two-back test for 30 minutes to induce fatigue in its participant pool before relying on a trail-making task to discern the effects of fatigue on cognition. The choice of a two-back task also affords the right balance between task workload and fatigability unlike the one-back or three-back protocols that are either too easy or challenging to the point of rapid saturation, an observation from our pilot efforts. Therefore, we co-opt this tested two-back protocol in our present investigation, but consider a singular task for 60 minutes to both induce fatigue and to evaluate WM across stimulation conditions.

We also espouse the vision of building a closed-loop solution for non-invasive neuromodulation in ER applications^18^. Current efforts with the use of electroencephalography show promise toward prescient state recognition^26,27^, however they remain impractical for field-use. Through this work we investigate the utility of heart rate variability (HRV) as an indicator of WM states across stimulation conditions. Prior studies have successfully demonstrated performance prediction when relying on HRV^28^, however the impact of tDCS and the robustness of these prediction paradigms remain unclear^29^. The neurovisceral integration model (NVIM) explores functional associations between cardiac vagal activity, and activation in the prefrontal cortex^30^, and could expand how we utilize HRV as a neurocognitive indicator. Therefore, the present study is focused on understanding the influence of anodal tDCS on WM performance during the fatiguing visuospatial two-back task. We consider a repeated measures, balanced experiment design with three conditions—anodal tDCS, sham tDCS, and control towards this goal. We hypothesize that anodal tDCS over the left DLPFC will prove an effective countermeasure to fatigue-related declines in WM capacity. Presently, we operationalize fatigue as a trait driven by the *time-on-task* effect and we characterize it by relying on a combination subjective self-reports and WM performance. Further, we look toward HRV as a means to understand changes in cognitive task performance using the NVIM.

## Methods

### Experiment design and methodology

**Figure 1:**
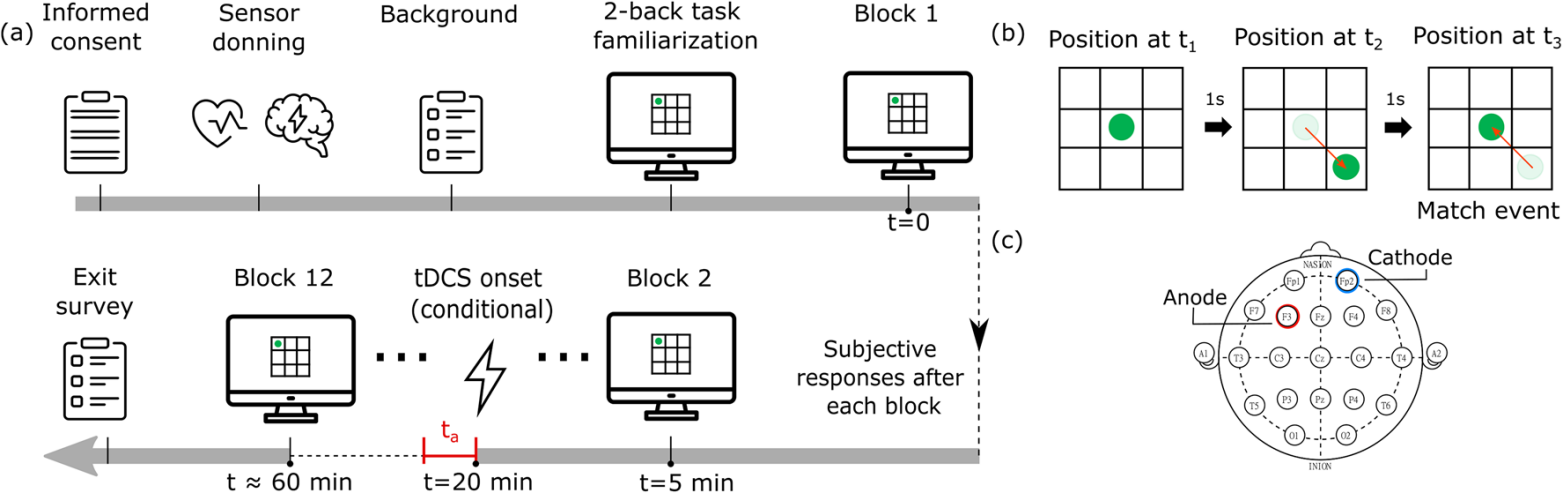
(**a**) Experiment protocol where $t_a$ represents the 10 min stimulation interval under the anodal tDCS condition, (**b**) schematic representation of a two-back match-event, and (**c**) tDCS electrode montage, cathode over the right supraorbital region (FP2), and anode over the left DLPFC (F3). Image created using *Inkscape 1.0.2-2*, https://inkscape.org/.

This study employed a repeated-measures, counterbalanced *Latin* square design^31^, with participants returning on separate days to complete a working memory exercise under three distinct conditions—control, anodal or sham tDCS. Participants were cast into three sex-balanced cohorts and the order in which each cohort was exposed to the treatment condition was based on the Latin square. Each session began with informed consent followed by subjective questionnaires on their mood (Profile of Mood States; POMS^32^) and sleepiness (Karolinska Sleepiness Scale; KSS^33^) levels (see Fig. 1a); before their first sessions participants also responded to a background questionnaire. The sessions lasted $\approx 60$ minutes each, and were divided into 12 blocks during which time participants completed a WM task. During anodal and sham tDCS conditions a researcher administered interventions at the start of the fifth block, i.e. approximately 20 minutes from the start of the session, while remaining outside the participant’s field of view. Prior investigations on performance deficits due to the *time-on-task* effect consider 20–30 min as the ideal window for fatigue onset^19,25^, and it is our goal to explore potential opportunities for intervention around this interval. Furthermore, participants were blinded to the stimulation condition (sham or anodal), but were aware of control sessions due to the absence of stimulation peripherals. Between each block, participants responded to a subjective questionnaire related to their levels of fatigue, effort and perceived discomfort, they were instructed to complete this as quickly as possible. The average block transition time was $\approx 15$ s. In addition, to reduce any anticipatory bias, participants were not informed of the precise duration of each block or experiment session.

### Participants

Fifty four participants were recruited for this study, only 32 (16 female) among them completed all three experiment sessions due to the constraints of the pandemic, i.e. 96 sessions across all treatment variables. The median age was $26 \in [18,~34]$ years. All participants were neurotypical and reported no adverse reaction history to tDCS, or treatments for neuropsychiatric and brain-related disorders. 29 out of the 32 participants in this study reported getting at least 6 hours of sleep in the days preceding each experiment session, with a median sleep quality rating of 7 on a ten point scale, where 0 indicated a state of “severe exhaustion” due to lack of sleep, and 10 indicated a state of being “extremely well rested”. Roughly $60\%$ (N = 19) of our participants reported a daily activity level of $4000-8000$ steps, with 3 reporting activity levels in excess of 10000 steps and the remaining below 4000. Participants reported a median score of 7 on a 10 point scale, with regards to their level of motivation to participate in the study, where 0 indicated “absolutely no motivation” to proceed, and 10 being “extremely motivated”. In addition, during recruitment, participants were subject to a list of exclusion criteria^34^ that included past neurological disorders, use of over the counter medication and caffeine habits. All experimental procedures were approved by Texas A&M University’s Institutional Review Board (IRB2019-1591DCR), and proceeded in accordance with a strict infection control plan and the Ethics Code of the American Psychological Association. Participants provided written informed consent before the start of each experiment and were reimbursed for their time.

### Working memory task

Participants were subject to an hour long visuospatial two-back WM test, while seated in front of a personal computer and provided a keyboard to record their responses. On the task, they tracked the position of a circle within a $3\times 3$ grid, if the position of the circle matched the one from two steps prior, they responded by pushing the space-bar (see Fig. 1b). The inter-stimulus-interval was 1 s with a persistence time of 900 ms. The two-back match probability ($p_{match}$) was 0.6; the interface served a fixed number (N = 94) of randomly timed match events within each 5 minute block. Before starting their first session, participants were introduced to the task and allowed to practice for a minimum of 5 minutes under a training mode to ensure that they understood task instructions. In this mode, the interface provided textual feedback on their response time and correctness (RED = incorrect, GREEN = correct). During the actual experiments this feedback was withheld; participants began each session on self-indicating their willingness to proceed after the practice period. The interface recorded every key-press and stimulus match event presented to the participant along with a time-stamp, response correctness tag (hit, miss, or false alarm), and response time (in ms).

### Transcranial direct current stimulation

A $1 \times 1$ tDCS device (Soterix Medical, NY, USA) was used in this study. The primary region of interest for WM was the left DLPFC, i.e. anode over F3 according to the 10-10 EEG system, with the right supra-orbital (r-SO) region or FP2 used as the reference location (see Fig. 1c)^35^. Participants were instrumented with stimulation peripherals during anodal and sham tDCS conditions only. Before each session participants were acquainted to the sensation of the stimulus. The current intensity was set to 1 mA, and the current density was $0.028~A/m2 ~(area = 5 \times 7~cm^2)$. Under anodal tDCS, the stimulation duration was 10 min at set point (1 mA); under sham tDCS, there was a ramp to the set point followed by a ramp to zero, lasting a total duration of $\approx 20$ s. The stimulation onset time was the same for both conditions—at the start of the fifth block, $\approx 20$ minutes from the start of each session. Stimulation onset time and condition were withheld from participants.

### Metrics

#### Working memory performance

Three performance measures, namely accuracy, specificity, and sensitivity, were used to assess task performance on the WM test employed in this study. Table 1 presents a description of each measure. For analysis, the data was grouped into five phases I (baseline), II, III (stimulation), IV and V (terminal), such that phase I to IV consisted of two blocks each, and V was made of three blocks, with each block lasting 5 minutes. This phase resolution was adopted—(1) to ensure that the two-block stimulation phase (i.e. blocks 5,  6 in phase III) was preserved uniquely in our comparisons, while ensuring block integrity across all phases; and (2) since block 12 was excluded from our analyses due to a self-reported anticipation-bias, where participants realized that the experiment was near its end (e.g. by counting the number of blocks) and returned to a state of alertness.

**Table 1:** Visuospatial two-back task performance metrics.

Performance measure	Description
Accuracy	$(TP+TN)/(P+N)$
Sensitivity	(*TP*)/(*P*)
Specificity	(*TN*)/(*N*)
Response delay (ms)	Time between stimulus and key-press

TP = **T**rue **P**ositives; P = all **P**ositive or *response selection* events.

TN = **T**rue **N**egatives; N = all **N**egative or *response inhibition* events.

#### Heart rate variability

Cardiac electrical activity was obtained from a chest-based electrocardiogram (ECG) probe and amplifier interface at 128 Hz (Actiheart 4, CamNTech, Inc., UK). The electrodes were positioned at the base of the sternum and over the left pectoralis minor muscle. The raw ECG signal was filtered for motion artifacts^36^, and corrected for ectopics with polynomial interpolation^37^. Subsequently, a peak detection algorithm was used to isolate the R peaks of the ECG signal^38^. The time between successive R–R peaks, i.e. the inter-beat-interval (IBI), was then derived from the processed peak signals. For subsequent analyses, we derived two representative statistics for every 5 min window in the raw IBI data, one in the time domain (RMSSD) and another in the frequency domain (LF-power; see Table 2). The measures were $min-max$ normalized across all three sessions, for each individual, before statistical analyses.

**Table 2:** Heart rate variability indices.

HRV measure	Description
RMSSD	**R**oot **M**ean **S**quare of the **S**um of **S**uccessive inter-beat interval (IBI) **D**ifferences
LF	Power density of the **L**ow **F**requency (0.04–0.15 Hz) component in the IBI spectrum

#### Subjective responses

Participants were subject to three one-point subjective questionnaires related to their levels of fatigue, expended effort and perceived discomfort during the brief transition interval ($\approx 10$ s) between each 5 minute experiment block. Participants rated each subjective attribute on a 10-point scale, where a score of 1 was “low or minimal”, and a score of 10 was rated “extreme or unbearable.” Besides the block transition questionnaires, participants were also subject to the POMS and KSS surveys before (PRE) and after (POST) each experiment session. We used an abridged version of the POMS survey with 39 questions across six emotive categories—tension-anxiety, depression-rejection, anger-hostility, vigor-activity, fatigue-inertia, and confusion-bewilderment^32^. Participants qualified each emotion on a 5-pt scale with 0 being “not at all”, and 4 being “extremely.” The score across all descriptors in a category was summed to generate a factor score; and this factor score was used to generate a composite mood disturbance score. The KSS reflected participant sleepiness levels ranging from 1 meaning “Extremely Alert” to 10 meaning “Extremely sleepy, can’t keep awake”.

#### Statistical analysis

The primary goal of the statistical analysis in this investigation was to explore the influence of experiment condition on performance, physiological and subjective responses at different time points (phase), and across both sexes during the WM exercise. Independent analysis techniques were applied for each dependent measure due to constraints in the data, but a common time scale was used across performance, HRV, and inter-block subjective responses, where the data was grouped into five phases. For the POMS and KSS, statistical comparisons were drawn between the PRE- and POST-experiment reports across each condition. Bonferroni corrections were applied where relevant to set the significance level at an appropriate threshold for multiple comparisons. For purposes of clarity, data in the figures are illustrated using the mean value and the standard error of the mean (S.E.M.); all graphs were created using Matplotlib 3.4.2^39^.

WM performance measures were not normally distributed, we relied on the non-parametric Friedman’s test to assess the effects of condition and sex (male, female) in each phase. Values that were more than three standard deviations from the mean were labeled outliers and excluded prior to analysis. Kendall’s W was used to determine the effect size. Significant outcomes on the Friedman’s test were then evaluated with pairwise Wilcoxon signed-rank tests. The HRV metrics introduced in Table 2 agreed with normality and sphericity constraints of the repeated measures ANOVA. Therefore, we performed a three-way repeated measures ANOVA to test the main and interaction effects of condition, phase, and sex on each metric. All two way interactions were analyzed to assess simple main effects and simple pairwise comparisons through t-tests. In addition, we compared the *baseline-subtracted* median accuracy scores between cohorts using a Kruskal-Wallis one-way analysis of variance test, and found no significant difference between them in any phase or condition (H-statistic $\in [0.46, 5.15]$; all *p* values$>0.053$), suggesting a balanced distribution of WM capacities across cohorts. Subjective inter-block responses were not normally distributed, and therefore analyzed using a similar approach to that on performance data. Analyses on the PRE- and POST-experiment mood and sleepiness surveys relied on simple paired t-tests. Where relevant, normality was assessed using the Shapiro-Wilk’s test, sphericity using Mauchly’s test for sphericity, and the homogeneity of variance among participants using the Levene test. All statistical analyses were performed in R using the *rstatix* and *tidyverse* packages.

## Results

### Working memory performance

**Figure 2:**
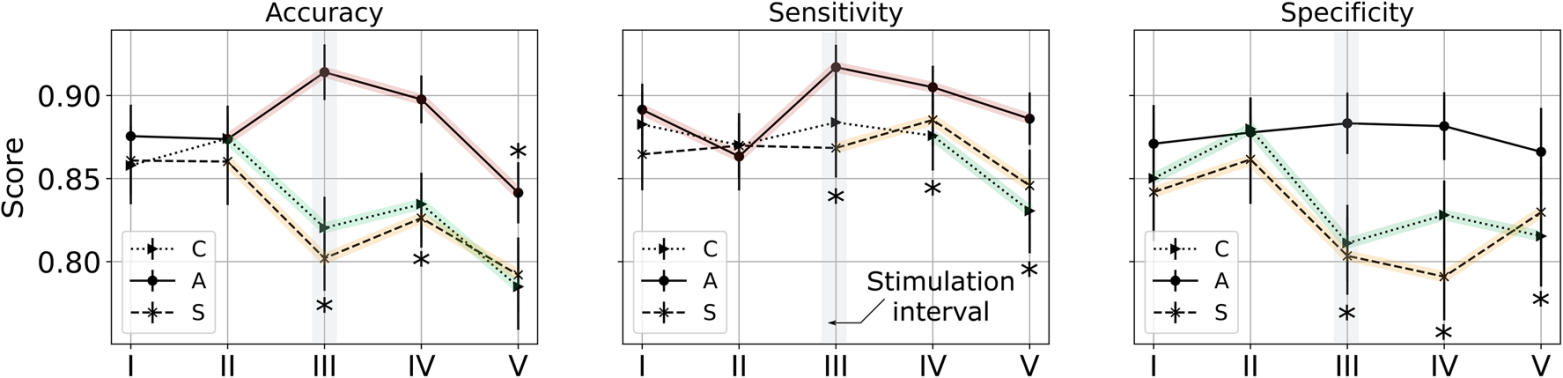
Trends in task performance for each phase as represented by the mean value and its standard error for each condition (control, anodal, and sham tDCS). The shaded segments represent *consecutive* time points where a statistically significant effect of time was found; the asterisk presents time points (phase) where a significant effect of condition was evidenced.

During baseline, i.e. phase I and through phase II, there was no effect of condition on any of the performance measures (all *p* values $> 0.2$). During stimulation and beyond, i.e. phase III to V, a significant effect of condition was observed on all three metrics (all $p < 0.0033; ~k_w \in [0.188, ~0.407]$). Post-hoc analyses revealed that during phases III, IV and V, all measures of performance were significantly better under anodal tDCS relative to sham tDCS or control (all $p < 0.0022$). A main effect of time was observed across all conditions for the sensitivity and accuracy measures. For specificity, this was true only under control and sham tDCS (all $p < 0.022; ~k_w \in [0.056, ~0.223]$). Overall, we note a decreasing trend in accuracy under sham tDCS and control. This decrease began from phase II, followed by a marginal improvement from phase III to IV, before declining further in phase V (all $p < 0.008$). Under anodal tDCS, we note an improvement in accuracy from phase II to III, and a gradual decrease across phases III and V (all $p < 0.00086$). Sensitivity under anodal tDCS exhibits a trend similar to accuracy across phase II and V (all $p < 0.027$). Under sham tDCS and control, sensitivity remained unaltered between phase I and III and phase I to IV respectively (all $p > 0.054$). However, in both conditions, we observed a decrease in the terminal phase (all $p < 0.048$). Specificity under sham tDCS and control was seen to improve in baseline and decrease from phase II to III, followed by contrasting trends across phase III and V (all $p < 0.007$; see Fig. 2). No effect of sex was found on any condition or time variable (all $p > 0.71$).

### Heart rate variability measures

**Figure 3:**
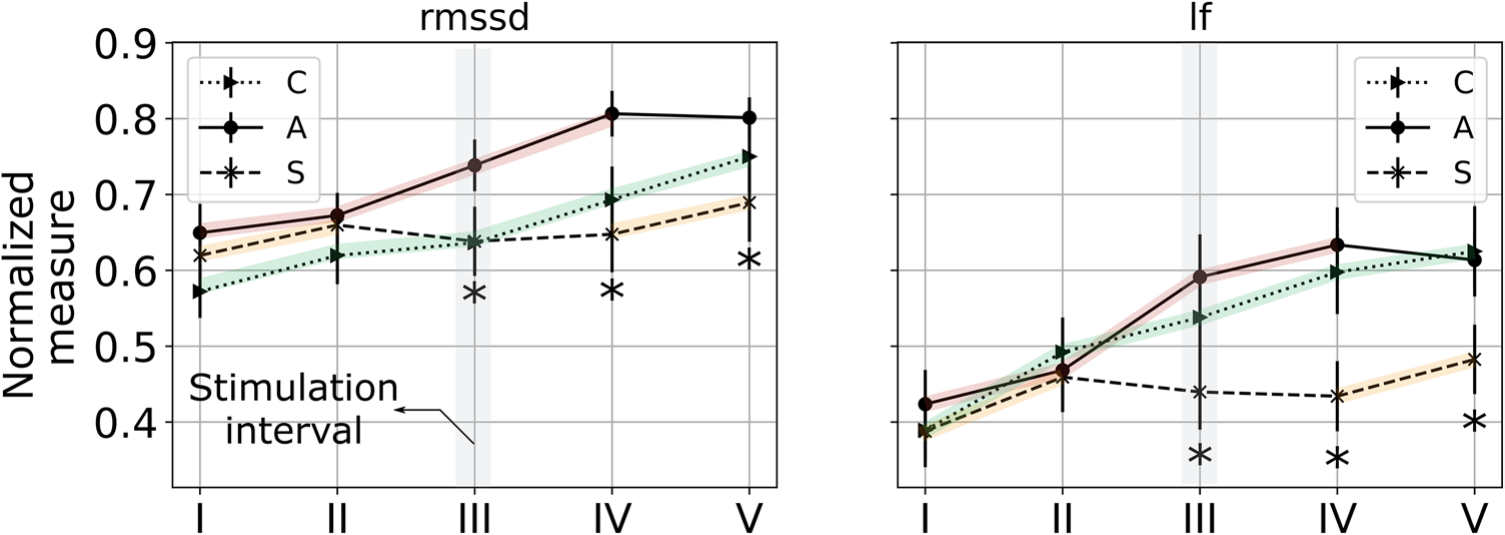
Mean and standard error of HRV features across the five phases: RMSSD (time-domain), LF-power (frequency-domain). The shaded segments represent *consecutive* time points where a statistically significant effect of time was found; the asterisk presents time points (phase) where a significant effect of condition was evidenced.

No effect of sex was found on any HRV measure considered (all $p > 0.89$). We found a significant main effect of time on the RMSSD values across all conditions (all $p < 0.0026;~\eta _g^2\in [0.16, ~0.23])$. Post-hoc comparisons revealed an increase in RMSSD across phase I and II under each condition (all $p < 0.007$). Under anodal tDCS, this increase in RMSSD was seen to persist until phase IV, followed by a plateau in phase V (all $p < 0.001$). Under sham tDCS, we observed that RMSSD remained relatively unchanged beyond phase II (all $p > 0.149$). Under control, we note an increasing trend in RMSSD, with statistically significant differences between phase III and IV, and phase IV and V (all $p < 0.019$). A main effect of condition was observed ($p< 0.0001; ~\eta _g^2= 0.14$), where in phases III and IV, we found that RMSSD under the anodal condition was significantly higher than that under control or sham tDCS ($p < 0.019$). For the LF-power measure, a significant main effect of time was apparent in all three conditions (all $p < 0.0016;~\eta _g^2\in [0.08, ~0.21]$). Post-hoc comparisons revealed an increase in LF power from phase I to II in all conditions (all $p < 0.041$). This increasing trend persisted under control and anodal tDCS until phase IV (all $p < 0.003$). Beyond phase IV, LF power under anodal tDCS was found to decrease in the terminal phase ($p = 0.002$), while remaining unchanged under the control condition ($p = 0.191$). Under sham tDCS LF-power was found to remain unchanged from phase II to IV (all $p > 0.076$), however in the terminal phase LF-power was elevated ($p = 0.041$); see Fig. 3.

**Figure 4:**
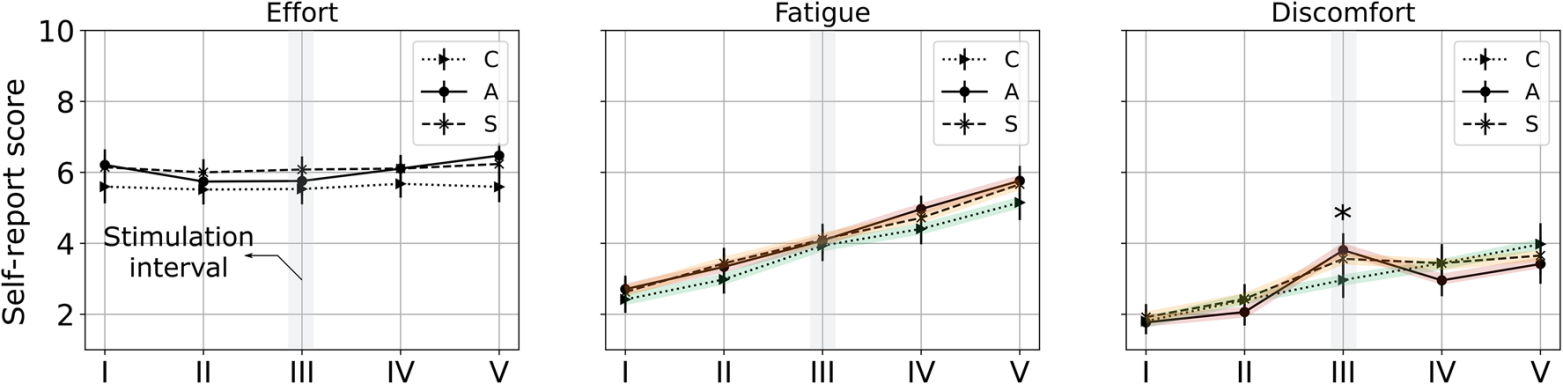
Perceived effort, fatigue, and discomfort scores as a function of time. The shaded segments represent *consecutive* time points where a statistically significant effect of time was found; the asterisk presents time points (phase) where a significant effect of condition was evidenced.

**Figure 5:**
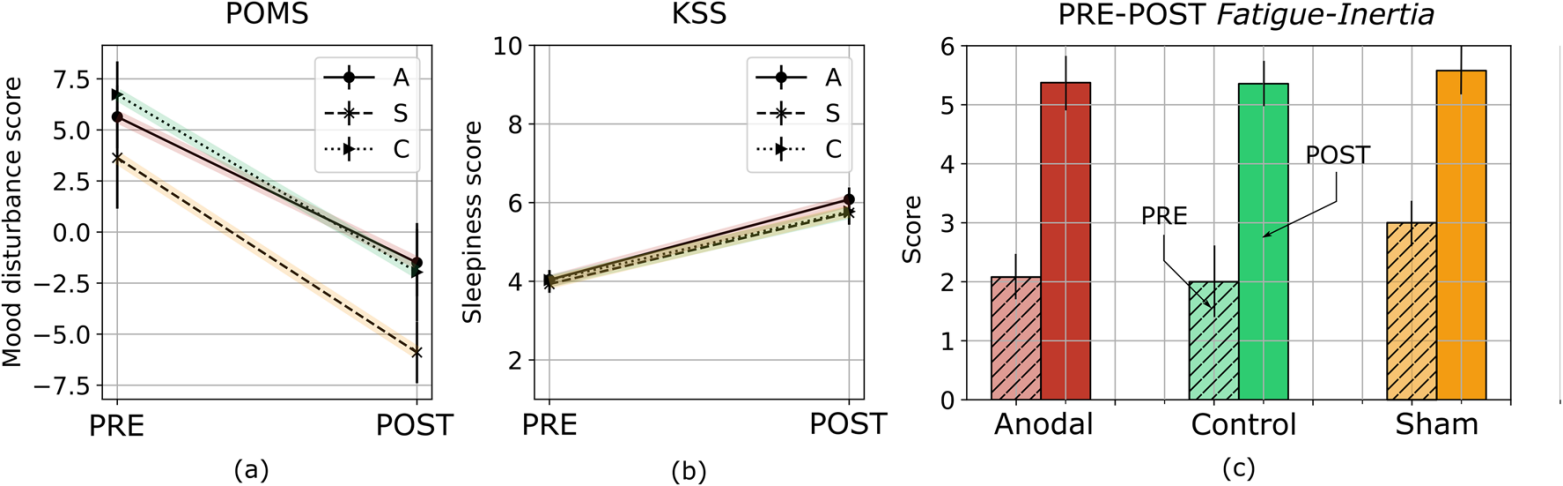
PRE- and POST- (**a**) mood disturbance and (**b**) sleepiness levels across all conditions as assessed by the Profile of Mood States survey and the Karolinska sleepiness scale. (**c**) PRE- and POST- *fatigue-inertia* scores from the POMS survey.

### Subjective responses

No effect of condition was found across the subjective dimensions of effort, and fatigue (all $p > 0.122$). A marginal effect of condition was evident for the discomfort measure where participants reported higher discomfort under sham, and anodal tDCS conditions relative to control during the stimulation interval, i.e. phase III ($p < 0.054$; see Fig. 4). A significant effect of time was observed across all three conditions for the discomfort and fatigue measures (all $p< 0.0001;~k_w \in [0.27, ~0.43]$), with participants reporting increased perceptions of fatigue and discomfort over time (all $p< 0.0022$). On the effort scores, a significant effect of time was reported only under the anodal tDCS condition (p= $0.027;~k_w = 0.08$). Post-hoc analyses revealed a marginal decrease of effort scores in phase III relative to phase I under this condition ($p= 0.054$). No effect of sex was found across the subjective responses (all $p > 0.561$). Furthermore, a significant difference was found in the PRE- and POST- mood disturbance and sleepiness scores across all conditions (all $p < 0.009$), with a mood disturbance decrement of $\approx 8.4$ points and sleepiness increment of $\approx 2.2$ points after the completion of each session. No effect of sex or condition was evident for either measure (all $p > 0.316$; see Fig. 5a, b).

### Power analysis

Thirty two participants completed this study (16 female), i.e. 96 sessions across all treatment variables. We employed a repeated measures design where participants were cast into three sex-balanced cohorts (10, 10, and 12) with the order in which they were exposed to each treatment condition (anodal, control or sham) counterbalanced through a Latin square. A power analysis was conducted to verify if our experiment had sufficient power to detect the effect sizes found significant in prior literature when employing a similar WM test, experiment design and stimulation protocol ($\eta ^2_p = 0.3$)^40^. Sample size estimation for a repeated measures ANOVA at a power level of 0.8, an error probability of 0.05 and partial eta-squared ($\eta ^2_p$) of 0.3 using the G*Power software^41^ suite revealed that a total sample size of 15 was required to test our hypothesis for the desired effect size. Given that we rely on non-parametric tests for significance in a subset of our variables, a $15\%$ rule of thumb^42^ increases the required sample size to 18 for the desired significance; 32 participants completed our experiments.

## Discussion

The present study explores the role of anodal tDCS as a countermeasure to the *time-on-task* (TOT) effect during a fatiguing visuospatial WM exercise. The TOT effect, we hypothesized, would result in decreasing WM capacity and an increase in the perceptions of fatigue when individuals are engaged in tasks with sustained cognitive demand for extended periods of time. For example, in the work by Mockel et al.^20^, on a 3 hour long *Simon* task^43^, perceptions of fatigue, and performance levels were shown to progressively deteriorate within the first hour of task and further so with time. Therefore, in some ways, the TOT effect is analogous to the human fatigue response, although the timescales for typical fatigue experiments are longer. Previous efforts have established the role of stimulation as a fatigue countermeasure for attention and response inhibition when relying on tasks relevant to those constructs, e.g. Go/No-Go tests, the Mackworth clock test, etc.^16,44^, indeed, investigations have also shown the efficacy of tDCS in improving WM in other conditions^45,46^. However, evidence that supports the use of tDCS as a countermeasure for TOT effects on WM remains unclear, in fact, antecedent results show a lack of improvement in WM capacity under fatiguing conditions with intervention at different current intensities and duration^16,17^. While the modes of fatigue onset remain disparate across these results, informed by prior work related to TOT-fatigue^24,25^, we employed a visuospatial two-back task to serve as both a *fatigue induction* mechanism and as a tool to evaluate WM. We compared performance, subjective and physiological responses, the influence of sex as a factor, and the effects of stimulation through a single-blind, repeated measures experiment, counterbalanced for learning across the three treatment conditions.

In both sexes, we found that anodal tDCS improved WM performance, beyond TOT-driven deficits that were otherwise evident across all performance measures (see Fig. 2). These improvements were found to last beyond the stimulation interval and enabled distinct changes that were unique to each performance measure for e.g., sensitivity improved under stimulation while specificity was maintained. However, no significant perceptual differences in self-reported effort or fatigue scores were noted between conditions. In general, participants indicated a marginal decrease in effort-scores under anodal tDCS and greater levels of discomfort during stimulation, as expected. Fatigue and discomfort reports were found to increase in a condition-agnostic manner over the time-course of the experiment, consistent with our expectations of the TOT effect, however, perceived effort remained similar throughout the hour long experiment, indicating participant motivation to stay on task. While mood and sleepiness changed with time, they were not significantly influenced by the condition variable.

The visuospatial two-back test, in its protracted format, demands both WM and sustained attention^47^. The central executive^48^ is responsible for encoding and updating an internal WM buffer, recognizing match events, and priming task-relevant actions. The brain regions essential to this activity include the prefrontal, motor, and visual cortices^49^. These areas enable substantial behavioral adaptation, attention or response inhibition, and learning during WM exercises^50,51^. However, with *time-on-task*, individuals invariably feel fatigued or otherwise depleted^19^. The onset of TOT-fatigue is marked by a decrease in neural activity across regions responsible for maintaining task-related behaviors, that are preempted by an increase in activation in regions peripheral to those networks^52^. These neurocognitive changes are supported by subjective fatigue scores, typically self-reported, or indirectly referenced from performance decrements^53^; in our protocol we rely on both modalities to operationalize TOT-fatigue. As reported, we found that subjective fatigue scores increased with time while PRE-POST surveys showed worsening mood after the completion of the experiment (see Fig. 5a, b). Specifically, a substantial and statistically significant increase was found in the *fatigue-inertia* emotive category (see Fig. 5c). According to the work by Schwartz et al.^54^, the minimally important clinically-relevant difference (MICD) indicating fatigue on the POMS survey was 5.6 points, roughly 1.1 points for each category, and the MICD on a 1-pt fatigue questionnaire, similar to the one used in this investigation, was 2.4. In our observations, changes in the POMS mood disturbance scores, the POMS *fatigue-inertia* scores and the inter-block 1-pt fatigue responses exceeded these thresholds with a mean decrement of $8.45\pm 0.98$ points, and increments of $3.077 \pm 0.35$ and $2.94 \pm 0.14$ points respectively, signaling the negative influence of sustained task performance on fatigue perception and reaffirming our TOT-fatigue hypothesis. Furthermore, this change was also reflected in performance behaviors as participants displayed a reduction in accuracy, sensitivity and specificity during both control and sham tDCS conditions beyond phase II, i.e. roughly 20 min into the experiment (see Fig. 2). Therefore, we reason that the observed changes in participant behaviors are driven by TOT-fatigue.

In light of the above evidence, the key finding in our work was affirmation that anodal tDCS at 1 mA for 10 min improved WM beyond TOT deficits. This improvement was seen both as the enhancement of WM capacity relative to baseline (accuracy, sensitivity), and as the preservation of performance levels over time (specificity; see Fig. 2). In addition, the effects were both concurrent and beyond transient, lasting for up to 20 min after the stimulation interval. Furthermore, we observed these changes in an experiment protocol, where intervention was provided after 20 min of sustained cognitive demand, which is noteworthy given mixed evidence thus far from studies that considered the online effects of tDCS on WM and contradictory observations of its effect on fatigue^13,14^. Furthermore, earlier investigations with offline or online stimulation for longer durations and at current intensities greater than 1 mA reported no positive effect on WM under fatiguing or fatigued conditions^16,17^. Additionally, we found no improvements in mood and sleepiness due to stimulation, contrary to some prior observations. Besides differences in the format of the WM task or stimulation parameters, we believe differences in the *fatigue induction* paradigm could be responsible for some of these divergent observations. For e.g., McIntire et al.^16^ explored fatigue driven by extended wakefulness, while our protocol demanded sustained attention for $\approx 70$ min. Therefore, the nature of fatigue on the task is a factor to consider in future investigations.

Curiously, we note that although performance levels improved under anodal tDCS, changes in the level of perceived effort and fatigue remained similar across conditions (see Fig. 4). A recurrent hypothesis in this domain attributes cognitive enhancements enabled by tDCS to changes in neural efficiency^55^, however subjective reports indicate that these “benefits” are mostly imperceptible, which demands consideration on how these intervention modes will be operationalized within human-centered systems. In addition, the self-reported fatigue scores appeared in contrast to those seen in the motor domain, where tDCS was shown to alleviate levels of perceived fatigue^56,57^. The reasons for this disparity are perhaps related to the fundamentally distinct neurocognitive footprint of muscular and cognitive fatigue^58^, regardless, larger studies with concurrent neuroimaging are necessary to unpack the neural mechanisms that underlie these distinctions, which remains the focus of our future efforts.

On performance improvements under anodal tDCS, we found that stimulation did not produce the same effect on sensitivity and specificity measures. During sham tDCS and control we discovered that sensitivity remained unchanged until the terminal phase, while specificity decreased beginning from phase III. Secondly, under anodal tDCS, sensitivity increased concomitant with stimulation, while specificity was maintained throughout the 60 min task. It is likely that these distinctions are due to the fundamental differences in the behaviors these measures capture. Sensitivity identifies response selection, in this context, it characterizes event recognition and the commission of a prepotent motor response, while specificity identifies response inhibition and reflects the ability to withhold motor impulses^59^. Bender et al. demonstrated that these are fundamentally distinct cognitive operations governed by functionally and structurally independent brain regions^60^, which may explain some of the observed differences. Studies also identify distinct cortical networks that underlie these processes, e.g. Rowe et al. showed the central role of frontoparietal networks, including the DLPFC, in response selection both when associated with WM and other tasks of willed action^61^; while others recognize the inferior frontal gyri (IFG), parieto-temporal junction, and supplementary motor areas as those essential toward inhibitory behaviors^62^. Filmer et al.^63^, when using similar stimulation parameters, reported augmentation in response selection during anodal tDCS of the left lateral prefrontal cortex, while other efforts point to the role of the right IFG in enhancing task-relevant response inhibition under tDCS^64^. Therefore, we reason that the non-focal nature of $1\times 1$ tDCS driven by the electrode size, current intensity and current density may be contributing to the differential impacts on response selection or inhibition behaviors observed in our investigation. Future investigations into the role of fatigue or *time-on-task* on these specific cognitive processes are necessary to shed light on the precise nature of their response and receptivity to stimulation.

HRV indexes the interaction between the central autonomic network and cardiac activity. The neurovisceral integration model posits that the main role of the prefrontal cortex during a WM task is toward attentional inhibition^30^. Empirical evidence points to a functional link between attentional inhibition mediated by the prefrontal cortex and vagal activity^65^. In our WM task, we expected that this would manifest as an increase in HRV during the early periods followed by a decrease due to TOT fatigue. However, HRV as indexed by RMSSD and LF-power increased under anodal tDCS until it plateaued in the terminal phase, while it appeared to increase throughout the experiment under control. In contrast, under sham tDCS, we found that the HRV indices increased through baseline before plateauing during the remainder of the experiment. Contextualized by performance data and subjective responses, we reason that HRV trends under sham or anodal tDCS agree with the expectations of the NVIM, however the control outcomes deviate from the model. It is possible that this deviation can be interpreted as the cumulative effect of fatigue and sustained WM demand on participants, with prior research indicating the linearly additive effects on HRV and similarly confounding observations^29^. Future investigations should consider HRV as an independent variable in investigating the influence of stimulation, which along with behavioral state dynamics and task state dynamics will serve as a crucial element in the development of fieldable closed-loop solutions for neuromodulation^66^.

## Limitations

We reported an anticipation bias in some participants during the terminal block, which required that we exclude it from our analyses, while this did not impact the trends observed in performance or subjective response, it remains a factor to consider in future study designs to avoid unanticipated influences on participant motivation while on task. Second, we do not adapt workload on the task to individual WM capacity, this appears as common practice in some earlier investigations on tDCS^67^, however, we reasoned that a prolonged task format would elicit a consistent TOT-fatigue response in our participants which was determined to be the case. Third, our participant blinding strategy was potentially inadequate—during the experiments, participants were blinded to sham or anodal tDCS, based on existing best practices^68^. The consistent discomfort scores between the two conditions indicates that our blinding between stimulation sessions was likely satisfactory, however, the absence of stimulation peripherals during control may have skewed participant experience during that condition. Fourth, we do not capture motivation or engagement levels during active task performance. We made this decision to avoid disruptions to the participant’s experience while on task, however, these measures could have improved how we understand the impact of tDCS on WM under fatigue^69^. Overall, our findings support the need for future investigations into the neural underpinnings that drive WM improvements, above and beyond the deficits induced by TOT-fatigue.

## Conclusions

Cognitive fatigue can have serious consequences in safety-critical domains such as ER. Our research showed that, under controlled conditions, WM can be enhanced beyond the influence of TOT-fatigue with the help of anodal tDCS. Further investigation is necessary to provide clarity on the specific neural origins of these changes and their perceptual relevance to the human in the loop. Moreover, future explorations must consider how WM enhancements under abstract conditions in the laboratory translate to real-world ER WM demands. Additionally, although our work points to the positive influence of stimulation on task output, subjective feelings of effort, fatigue, and discomfort were not seen to benefit from neuromodulation, which demands consideration on “how” these technologies will be operationalized within human-centered, high-risk socio-technical systems.

## Data Availability

All experimental procedures were approved by Texas A&M University’s Institutional Review Board (IRB2019-1591DCR), and proceeded in accordance with the Ethics Code of the American Psychological Association. Data will be made available upon reasonable request to the authors.
